# Vertebrate cardiac regeneration: evolutionary and developmental perspectives

**DOI:** 10.1186/s13619-020-00068-y

**Published:** 2021-03-01

**Authors:** Stephen Cutie, Guo N. Huang

**Affiliations:** 1 Cardiovascular Research Institute and Department of Physiology, University of California, San Francisco, San Francisco, CA 94158 USA; 2 Eli and Edythe Broad Center for Regeneration Medicine and Stem Cell Research, University of California, San Francisco, San Francisco, CA 94158 USA

**Keywords:** Heart, Regeneration, Development, Evolution, Cardiomyocyte proliferation, Cell cycle arrest, Polyploidization, Endothermy, Inflammation

## Abstract

Cardiac regeneration is an ancestral trait in vertebrates that is lost both as more recent vertebrate lineages evolved to adapt to new environments and selective pressures, and as members of certain species developmentally progress towards their adult forms. While higher vertebrates like humans and rodents resolve cardiac injury with permanent fibrosis and loss of cardiac output as adults, neonates of these same species can fully regenerate heart structure and function after injury – as can adult lower vertebrates like many teleost fish and urodele amphibians. Recent research has elucidated several broad factors hypothesized to contribute to this loss of cardiac regenerative potential both evolutionarily and developmentally: an oxygen-rich environment, vertebrate thermogenesis, a complex adaptive immune system, and cancer risk trade-offs. In this review, we discuss the evidence for these hypotheses as well as the cellular participators and molecular regulators by which they act to govern heart regeneration in vertebrates.

## Background

### Cardiac regeneration in vertebrates

#### Lower vertebrates that can regenerate myocardium as adults

##### Teleost fish

Unlike humans and rodents, zebrafish are able to fully regenerate the myocardium as adults. Resection of up to 20% of the *Danio rerio* ventricle induces a robust proliferative response in the myocardium by 14 days post-injury and temporary fibrosis that resolves with complete regeneration and no scar by 60 days post-injury (Poss 2002). Lineage-tracing confirmed that zebrafish cardiac regeneration occurs via dedifferentiation and proliferation of pre-existing cardiomyocytes (CMs) after injury (Jopling et al. 2010; Kikuchi et al. 2010). Intriguingly, this complete cardiac regenerative potential in the zebrafish can be blunted by physiological stress, (Jazwinska and Sallin 2016) inhibition of vitamin D signaling, (Han et al. 2019) and artificial polyploidization of the myocardium (González-Rosa et al. 2018). Additionally, like their zebrafish relatives, the adult giant danio *Danio aequipinnatus* can fully regenerate myocardium after cautery injury, clearing all local necrosis and fibrosis by 60 days post-injury (Lafontant et al. 2012).

However, unlike zebrafish and danios, adult *Oryzias latipes* cannot regenerate after cardiac injury. Instead, ventricular resection results in minimal induction of CM proliferation and the formation of a permanent fibrotic scar (Ito et al. 2014). Delayed neutrophil clearance and reduced macrophage recruitment contribute to medaka’s inability to regenerate (Lai et al. 2017).

##### Newt

Resection of up to 10% of the *Notophthalmus viridescens* ventricle induces myocardial cell proliferation and resolves as complete ventricular regeneration by 70 days post-injury (Witman et al. 2011). CM proliferation of the predominantly mononuclear diploid variety is observed during newt cardiac regeneration (Oberpriller et al. 1988; Oberpriller and Oberpriller 1974). Newt CM plasticity even allows CMs transplanted into regenerating limb buds to transdifferentiate and express skeletal muscle and chondrocyte markers (Laube et al. 2006). The gene expression profile of the regenerating newt heart differs from regenerative expression profiles in other organs (Mercer et al. 2012).

##### Axolotl

In the adult *Ambystoma mexicanum*, both ventricular resection (Cano-Martínez et al. 2010) and cryoinjury(Godwin et al. 2017) of the myocardium induce CM proliferation and complete cardiac regeneration and restoration of heart function within 90 days post-injury, without permanent fibrosis. Notably, successful heart regeneration in the axolotl depends not only on the induction of CM proliferation after injury, but also on macrophage activity. Upon macrophage depletion, myofibroblasts induce excessive collagen deposition that results in permanent fibrosis (Godwin et al. 2017).

##### Frog

Evidence exists of at least partial myocardial regeneration in adult frogs. Early work in *Rana temporaria* observed a partial regenerative response after crush injury of the ventricle, with a post-injury induction of CM proliferation (Rumyantsev 1973) and subsequent sinus rhythm restoration to nearly 80% of animals within 200 days post-injury (Rumyantsev 1961). Furthermore, adult 1-year-old *Xenopus tropicalis* hearts mount a robust CM proliferative response to 10% apical resection that fully regenerates the lost myocardium nearly scar-free by 30 days after injury (Liao et al. 2017). Conversely, other research in *X. laevis* indicates that while tadpoles induce CM proliferation after oxidative damage (Jewhurst and McLaughlin 2019) and also mount a complete regenerative response after resection, post-metamorphosis 6-month old juveniles and 5-year-old adults can only mount a partial regenerative response to comparable apical resection (Marshall et al. 2019; Marshall et al. 2017). While this discrepancy between *X. tropicalis* and *X. laevis* may be explained by the different ages of the resected adults, it may also be explained by ploidy differences between the two species: *X. tropicalis* has a diploid genome with mostly mononuclear CMs, but *X. laevis* has a pseudotetraploid genome with mostly tetraploid CMs (Marshall et al. 2018). Further work may shed more light about this differential regenerative potential. It is not unprecedented for related species to have radically different cardiac regenerative capacities, as with zebrafish and medaka (Ito et al. 2014).

#### Vertebrates with potential or partial CM proliferative response as adults

Lizard: Unlike the less than 1% baseline CM proliferation rate in rodents and humans, baseline CM proliferation in the leopard gecko *Eublepharis macularius* is comparable to zebrafish at approximately 10% (Jacyniak and Vickaryous 2018). In adult *Agama caucasica* and *Lacerta armeniaca* lizards, cautery injury to the ventricular myocardium induces a proliferative response from nearby CMs around the injury site (Rumyantsev 1991). Additionally, puncture injury to the ventricle of the leopard gecko *Eublepharis macularius* induces proliferation of both CMs and non-CM epicardial cells that restore the pre-injury architecture of the wound within 14 day (Jacyniak and Vickaryous 2020). It remains unknown whether this lizard regenerative capacity extends to larger cardiac injuries such as ventricular resection and cryoinjury, as it does in zebrafish.

Bat: While apical resection or myocardial infarction have yet to be induced in bats, their cardiac regenerative potential may be higher than that of humans and rodents. Inversion experiments may have caused mechanical damage to CMs as indicated by increased creatine kinase levels and increased myocardial weight, but increased CM cell cycle activity and cardiac remodeling suggested regenerative potential of the bat *Eidolon helvum (*Ashaolu and Ajao 2014*)*.

#### Vertebrates that cannot regenerate myocardium as adults, but can transiently as neonates or embryos

##### Mouse

While adult *Mus musculus* are incapable of myocardial regeneration after cardiac injury, complete cardiac regeneration without permanent fibrosis is possible during embryonic and neonatal stages (Porrello et al. 2011; Porrello et al. 2013). Resection of ~ 15% of the ventricular apex in P0 mice induces cardiomyocyte (CM) proliferation that regenerates the lost myocardium without evidence of permanent fibrosis or compromised cardiac function by 21 days post-resection (Porrello et al. 2011). Similarly, ischemic cardiac damage caused by permanent ligation of the left anterior descending coronary artery (LAD) in P1 mice resolves as fully-regenerated cardiac muscle without evidence of fibrosis by 21 days post-ligation (Porrello et al. 2013). In both cases, CMs in the regenerated myocardium came from pre-existing CMs that were induced to dedifferentiate and proliferate after injury, and the neonatal regenerative response was lost by P7 (Porrello et al. 2011; Porrello et al. 2013). This closure of the murine neonatal regenerative window between birth and P7 coincides with the developmental polyploidization of the neonatal myocardium during the first week of life, during which > 90% of CMs binucleate and permanently exit the cell cycle (Soonpaa et al. 1996; Cao et al. 2019). Thus, CM polyploidization is proposed as a major barrier to cardiac regeneration in mice, supported by observations that mouse strains with higher percentages of mononuclear diploid CMs induce more CM proliferation after cardiac injury and are left with smaller fibrotic scars (Patterson et al. 2017). Interestingly, while mononuclear diploid CMs proliferate at greater rates than binuclear polyploid CMs in vivo, both CM types proliferate at similar rates in vitro when co-cultured with neonatal rat ventricular myocytes or embryonic mouse CMs (Wang et al. 2017).

##### Rat

As with mice, a transient neonatal cardiac regenerative response has been observed in *Rattus norvegicus*. In P1 rats, 18% apical resection induced myocardial regeneration including robust, uniform troponin I deposition and Connexin 43 expression, as well as restoration of baseline cardiac function and perfusion by 60 days post-injury (Zogbi et al. 2014). However, this robust regenerative response was lost by P7, in which permanent apical scar tissue formed and cardiac function did not fully recover (Zogbi et al. 2014). Neonatal rats have also been observed to mount a regenerative response to burn lesions made in the myocardium (Robledo 1956).

##### Pig

Myocardial infarction induces a robust but transient regenerative response in *Sus domesticus*. Cardiac injury in P2 pigs induces CM proliferation that regenerates the lost myocardium and restores cardiac function without permanent fibrosis by 12 weeks post-injury.(Ye et al. 2018) P1 pigs fully regenerate from myocardial infarction even more rapidly, within 30 days (Zhu et al. 2018). However, this porcine regenerative response is progressively lost by P14 (Ye et al. 2018; Zhu et al. 2018). P3 pigs maintain substantial fibrosis at 30 days post-injury (Zhu et al. 2018) and fibrosis is still observed at 12 weeks post-injury,(Ye et al. 2018) suggesting that the porcine neonatal regenerative window closes very soon after P2.

##### Human

Cardiac regeneration has been anecdotally inferred in neonatal humans. There are documented cases of infants suffering massive myocardial infarctions shortly after birth and yet surviving without apparent long-term deficits in cardiac function (Boulton et al. 1991; Saker et al. 1997; Murugan et al. 2002; Cesna et al. 2013). One newborn that suffered a severe infarction at birth due to coronary artery occlusion showed normal cardiac function and morphology at 1 year old (Haubner et al. 2016). However, post-infarct CM proliferation induction in adult humans is limited (Beltrami et al. 2001). It remains to be established when cardiac regenerative potential is lost in humans.

##### Bird

Avian cardiac regenerative capacity has not been as thoroughly investigated as mammalian heart regeneration, but work in *Gallus domesticus* indicates that burn lesions in the myocardium induce permanent fibrosis in 18-day old chicks, but resolve as regeneration in 3- and 5-day old chicks (Rumyantsev 1991; Novikov and Khloponin 1982). This is consistent with birds having a cardiac regenerative window that closes soon after birth, as rodents do.

A summary of cardiac regenerative potential in various vertebrate species is presented in Table 1.

**Table 1:** Summary table of known cardiac regenerative potential among vertebrate species

Species	Adult cardiac regeneration	Partial regenerative response	Only transient cardiac regeneration
Zebrafish (*D. rerio*)(Poss 2002; Jopling et al. 2010; Kikuchi et al. 2010)	✔□		
Giant Danio (*D. aequipinnatus*)(Lafontant et al. 2012)	✔□		
Newt (*N. viridescens*)(Witman et al. 2011; Oberpriller et al. 1988; Oberpriller and Oberpriller 1974)	✔□		
Axolotl (*A. mexicanum*)(Cano-Martínez et al. 2010; Godwin et al. 2017)	✔□		
Western Clawed Frog (*X. tropicalis*)(Liao et al. 2017)	✔□		
African Clawed Frog (*X. laevis*)(Jewhurst and McLaughlin 2019; Marshall et al. 2019; Marshall et al. 2017)			✔□
Caucasian Agama (*A. caucasica* )(Rumyantsev 1991)		✔□	
Armenian Lizard (*L. armeniaca*)(Rumyantsev 1991)		✔□	
Leopard Gecko (*E. macularius*)(Jacyniak and Vickaryous 2018; Jacyniak and Vickaryous 2020)		✔□	
Chicken (*G. domesticus*)(Rumyantsev 1991; Novikov and Khloponin 1982)			✔□
Straw-Colored Fruit Bat (*E. helvum*)(Ashaolu and Ajao 2014)		✔□	
Pig (*S. domesticus*)(Ye et al. 2018; Zhu et al. 2018)			✔□
Rat (*R. norvegicus*)(Zogbi et al. 2014; Robledo 1956)			✔□
Mouse (*M. musculus*)(Porrello et al. 2011; Porrello et al. 2013; Soonpaa et al. 1996; Cao et al. 2019)			✔□
Human (*H. sapiens*)(Boulton et al. 1991; Saker et al. 1997; Murugan et al. 2002; Cesna et al. 2013; Haubner et al. 2016)			✔□

## Current hypotheses about the evolutionary drivers of cardiac regenerative potential loss in adult mammals

### Oxygen environment

Transition to an oxygen-rich postnatal environment has been proposed to induce CM cell-cycle arrest and cardiac regenerative potential loss (Puente et al. 2014). CM oxidative capacity rises during early postnatal development as fetal CMs shift from glycolysis as their principal energy source to neonatal CMs which rely primarily on fatty acid β-oxidation, coinciding with neonatal CM terminal differentiation (Lopaschuk and Jaswal 2010). Indeed, chemically inhibiting this developmental transition to fatty acid oxidation with etomoxir delayed CM cell-cycle exit and polyploidization (Cao et al. 2019). This shift from glycolysis to oxidative metabolism has also been observed in neonatal rabbit CMs (Lopaschuk et al. 1991). Paralleling the closure of the neonatal mouse cardiac regenerative window, reactive oxygen species and the corresponding DNA damage increase in mouse CMs throughout the first week of life (Puente et al. 2014). Scavenging reactive oxygen species from CMs delayed their postnatal cell-cycle exit and increased the percentage of mononuclear CMs, while augmenting reactive oxygen species accelerated CM cell cycle arrest (Puente et al. 2014). Additionally, chronic hypoxia alleviates oxidative damage and induces CM mitosis in adult mice and improves regeneration following myocardial infarction in adult mice (Nakada et al. 2017). Developmental stage of CMs may play a role in their hypoxia sensitivity: while hypoxia increased primary neonatal rat CM proliferation in vitro, it decreased proliferation of fetal rat CMs *in vitro (*Sun et al. 2019 *)*. While these evidence support the role of an oxygen-rich postnatal environment in promoting CM cell-cycle withdrawal, studies in precocial mammals such as sheep demonstrated that their CMs almost complete polyploidization and permanent cell-cycle arrest before birth, suggesting the existence of major physiological triggers other than oxygen to shut down CM proliferative potential (Jonker et al. 2015).

### Endothermy acquisition

Vertebrate thermogenic capability appears inversely correlated with cardiac regenerative potential. That is to say, tissue regeneration may generally be an ancestral vertebrate trait that was lost as adaptations like endothermy developed (Goss 1992). Much as neonatal mammals display increased cardiac regenerative potential compared to adults, neonates are also less able to thermoregulate than adults (Tourneux et al. 2009). Likewise, unlike regenerative species like zebrafish and newts, non-regenerative humans and rodents can efficiently thermoregulate as adults (Ivanov 2006). While the direct effects of endothermy on cardiac regeneration remain to be explored, hypothermia has been observed to enhance brain regeneration after stroke, corroborating an inverse relationship between thermogenesis and regeneration (Yenari and Han 2013).

Furthermore, a recently phylogenetic analysis of 41 vertebrate species showed that diploid CM constitution of the myocardium, a proxy of cardiac regenerative potential, correlates inversely with standard metabolic rate – the metabolic rate of any organism once the effects of organism size are factored out, as described by Kleiber’s Law (Hirose et al. 2019). Lower vertebrates with high cardiac regenerative potential and mostly diploid mononuclear CMs – zebrafish, newts, and reptiles – have standard metabolic rates an order of magnitude lower than those of endothermic eutherian mammals like rodents and humans (Makarieva et al. 2008; Schmidt-Nielsen et al. 1980). Increasing CM ploidy during vertebrate evolution paralleled their transition from ectothermy to endothermy. Furthermore, mammalian body temperature – a key determinant of metabolic rate in mammals – also correlates inversely with mammalian diploid CM percentage (Hirose et al. 2019). Altogether, these results suggest that physiological changes during the acquisition of endothermy in both development and evolution may induce CM polyploidization and cardiac regenerative potential loss.

### Immune response

The development of robust inflammatory responses and a complex adaptive immune system in ontogeny and phylogeny parallels the decline of tissue regenerative potential (Mescher and Neff 2005; Aurora and Olson 2014). It has been proposed that efficient immune action is a trade-off for regenerative capacity (Zhao et al. 2016). For instance, salamanders and frogs are both amphibians with higher cardiac regenerative potential than mammals as adults – however, only salamanders can fully regenerate limbs as adults (Witman et al. 2011; Cano-Martínez et al. 2010; Liao et al. 2017). Notably, the immune response in salamanders is more primitive, less specific, slower-onset, and less adaptive than that in the less-regenerative frogs (Aurora and Olson 2014). Even in frogs, loss of limb regenerative potential in *Xenopus* larvae coincides with the maturation of the adaptive immune system from an ancestral state to a mammalian-like immune system during metamorphosis (King et al. 2012). In mammals as well, developmental maturation of the immune system accompanies the loss of fetal scarless regeneration (Mescher and Neff 2005). Indeed, ablation of CD4^+^ T-cells – a critical player in the adaptive immune system enriched in non-regenerative P8 hearts compared to regenerative P3 hearts – increases CM proliferation and ameliorates fibrosis after apical resection (Li et al. 2020a).

Unlike the adaptive mammalian immune system, there is mounting evidence that the early innate immune response is a critical determinant of cardiac regenerative potential. Certain elements of the unresolved post-injury inflammatory response in adult mice contribute to their inability to regenerate, as inhibition of TLR2(Arslan et al. 2010) and TLR4 (Oyama et al. 2004; Timmers et al. 2008) and early resolution of the immune response at the infarct site (Kain et al. 2016; Vandervelde et al. 2006) improves cardiac regeneration in adults. Although acute inflammation after cardiac injury in adult mice fails to induce a regenerative response, the acute inflammatory response in P1 mice is critical for their cardiac regenerative response (Han et al. 2015). Specifically, macrophage depletion compromises cardiac regenerative capacity in P1 mice after, with macrophage-depleted neonates failing to induce neoangiogenesis and forming permanent fibrotic scars after myocardial infarction (Aurora et al. 2014). Furthermore, regenerative M2 macrophages in the P1 upregulated expression of soluble factors supporting myogenic differentiation and growth – specifically IGF1, TGFβ, activin-A, and Arg1 – that non-regenerative M1 macrophages in the P14 do not,(Aurora et al. 2014) corroborating the observation that regenerative M2 macrophages in P1 are predominantly of embryonic origin and promote CM proliferation and angiogenesis with minimal inflammation while non-regenerative M1 macrophages in P14 are monocyte-derived and invade the heart after injury to promote inflammation.(Lavine et al. 2014) The four aforementioned factors promote myogenesis, oligodendrogenesis, and myelinogenesis; and suppress inflammation.(Saclier et al. 2013; Miron et al. 2013; Pesce et al. 2009) The developmental transition from M2 to M1 may also play a role in the developmental loss of regenerative potential in other organs as well. Intriguingly, CCR2 inhibition in the adult heart improved neoangiogenesis and reduced inflammation after injury by blocking post-injury monocyte recruitment to the heart and preserving embryonic macrophage activity (Lavine et al. 2014).

The importance of the early immune response during cardiac regeneration also extends to fish. Inhibition of the early post-resection immune response via glucocorticoids in zebrafish increases collagen deposition, reduces angiogenesis, disrupts heart regeneration (Huang et al. 2013). Similarly, medaka’s inability to regenerate after ventricular resection may result from its delayed early immune response compared to zebrafish (Lai et al. 2017).

### Cancer prevention

It has been postulated that during evolution, mammals lost regenerative potential as a trade-off for cancer protection (Pomerantz and Blau 2013). This balancing act between regenerative potential and cancer risk has been mediated by tumor suppressor genes that reduce the oncogenic risks of stem cells but inhibit proliferation and regenerative potential. For example, while the ancestral gene in the p53 family of tumor suppressors arose in sea anemones to protect the germline from DNA damage, p53 itself and its explicit somatic tumor-suppressor function arose in early vertebrates like cartilaginous fish that first developed regenerative populations of somatic stem and progenitor cells that made vertebrates more susceptible to cancers (Belyi et al. 2010). As natural selection has selected for tighter controls against cancer risk in vertebrates, it has done so at the cost of stem cell proliferative capacity (Greaves 2007). Perhaps unsurprisingly, vertebrate regenerative potential also declines with age as age-associated DNA damage and cancer risks accumulate (Seifert and Voss 2013).

This trade-off between regenerative potential and cancer risk is evident in the mammalian heart. While both cancer risk and regenerative capacity vary by organ, both are low in the adult mammalian heart. The permanent cell-cycle exit that prevents CMs from proliferating in the adult heart may also explain the rarity of CM tumors. Furthermore, overexpression of certain oncogenes has been found to increase adult CM proliferation. Meis1 (Myeloid ecotropic viral integration site 1) promotes glycolysis in hematopoietic stem cells, is upregulated in various cancers, and is naturally downregulated in the neonatal heart as CMs exit the cell cycle; fetal Meis1 suppression pushes fetal CMs from glycolysis to oxidative phosphorylation prematurely (Lindgren et al. 2019). Likewise, oncogene and NRG1 co-receptor ERBB2 overexpression in juvenile and adult mice resulted in cardiomegaly & increased CM proliferation, and transient ERBB2 induction post-MI improves adult cardiac regeneration (D’Uva et al. 2015).

## New players: cellular participators & molecular regulators

### Nuclear receptors

#### Thyroid hormone receptor

Current data suggest an inhibitory role played by TH in the developmental control of neonatal CM proliferative potential. Reduced ventricular thyroid hormone signaling, low ventricular T3 and increased ventricular T4 in both ischemic and dilated cardiomyopathy likely due to reduced D2 deiodinase (Li et al. 2020b; Gil-Cayuela et al. 2017; Gil-Cayuela et al. 2018). Thyroid hormones regulate energy metabolism and are suspected drivers of the evolutionary ectotherm-to-endotherm transition (Hulbert 2000; Little and Seebacher 2014; Buffenstein et al. 2001). Recent evidence in vertebrates implicates thyroid hormones (THs) in promoting CM binucleation and suppressing CM proliferative potential (Hirose et al. 2019). Serum TH levels are also substantially lower in newts and zebrafish than in non-regenerative mammals, rising soon after birth in neonatal mice (Liversage and Korneluk 1978; Chang et al. 2012). Inhibition of TH activity in neonatal mice – either genetically or chemically through TH inhibitor propylthiouracil – increases mononuclear CM percentage and CM proliferation.(Hirose et al. 2019) CM-specific inhibition of TH signaling also enhances cardiac contractile functions and reduces fibrosis post-MI in adult mice. Whereas in adult zebrafish, exogenous TH suppresses CM proliferation and cardiac regeneration after resection.

#### Vitamin D receptor

Vitamin D is a steroid hormone precursor that can bind to nuclear vitamin D receptors (VDRs) and regulate downstream gene expression (Bikle and Vitamin 2014). CM-specific deletion of VDR promotes cardiac hypertrophy in mice.(Chen et al. 2011) VDR activation reduces expression of both PCNA protein levels and *c-myc* and inhibits proliferation in neonatal rat CM culture (O’Connell et al. 1997). Vitamin D analogs have been reported to inhibit proliferation in mammalian primary CMs and similar cell lines, (Hlaing et al. 2014; Nibbelink et al. 2007; Cutie et al. 2020) but these effects may not be conserved across all vertebrates. In zebrafish, vitamin D analogs increase CM proliferation in embryos and VDR-inhibitor PS121912 significantly decreases zebrafish CM proliferation (Han et al. 2019). VDR agonists also stimulate CM proliferation in adult zebrafish during heart regeneration, while VDR suppression decreases CM proliferation during regeneration (Han et al. 2019). Even in rodents, while VDR activation suppresses in vitro proliferation in primary P1 mouse CMs, whether it exerts anti-proliferative effects on P7 is less clear (Han et al. 2019; Cutie et al. 2020). The downregulation of VDR in the heart between birth and P7 may explain differential sensitivity of P7 CMs to VDR activation (O’Meara et al. 2015).

#### Glucocorticoid receptor

Glucocorticoids are stress hormones produced by the adrenal glands that bind to the glucocorticoid receptor (GR) in nearly all organs in the body.(Oakley and Cidlowski 2013; Evans 1988; Rog-Zielinska et al. 2013) They inhibit the cell cycle primarily through the GR, which acts as a transcription factor after binding (Crochemore et al. 2002; Smith et al. 2000). CM-specific deletion of GR promotes cardiac hypertrophy(Oakley and Cidlowski 2013; Cruz-Topete et al. 2019) while GR activation inhibits neonatal rat CM proliferation and increases CM binucleation (Gay et al. 2015; Gay et al. 2016). GR activation also promotes neonatal CM hypertrophy, inhibits neonatal CM proliferation, and increases CM binucleation through epigenetic repression of Cyclin D2 gene (Gay et al. 2015; Gay et al. 2016; Whitehurst et al. 1999). Additionally, both exogenous GR agonist exposure(Huang et al. 2013) and crowding-induced stress through GR activation impair regeneration in the zebrafish heart.(Jazwinska and Sallin 2016) sex-specific cardiac pathology after GR deletion implying that GR regulates CM calcium responses differentially based on sex (Cruz-Topete et al. 2019).

### Metabolic regulators

#### PKM2

Pyruvate kinase muscle isozyme 2 (Pkm2) developmental downregulation in CMs coincides with the closure of the rodent heart regenerative window. It reduces oxidative stress by promoting glycolysis and reducing pyruvate kinase activity. Its overexpression promotes CM proliferation both in the post-mitotic adult heart, and post-MI heart, while its downregulation in the embryo reduced heart size (Magadum et al. 2020).

#### PDK4

Not only can neonatal CM cell cycle arrest be delayed via inhibition of fatty acid oxidation, but inhibition of PDK4 (pyruvate dehydrogenase kinase 4) shifted CMs from hypertrophy to hyperplasia by shifting them from fatty acid oxidation towards glycolysis (Cao et al. 2019; Cardoso et al. 2020).

### Cell cycle progression

#### ECT2

Ect2 generates guanosine triphosphate during contractile ring assembly & cytokinesis initiation; its loss of function in ZF promotes CM polyploidization (González-Rosa et al. 2018). Likewise, Ect2 loss in neonatal CMs enhances developmental binucleation of the myocardium and downregulates proliferative pathways like E2f target genes,(Windmueller et al. 2020) whereas increasing neonatal Ect2 activity via chemical inhibition of β-adrenergic signaling conversely increases both total CM number and the fraction of mononuclear CMs (Liu et al. 2019). This reduced CM ploidy and increased CM endowment phenotype persists into adulthood, enabling superior functional recovery of the heart after myocardial infarction (Liu et al. 2019).

#### HOXB13

Upon dephosphorylation by calcineurin, Hoxb13 localizes to the cardiomyocyte nucleus during developmental cell cycle arrest and promotes the shift from hyperplastic to hypertrophic CM growth. CM-specific deletion of Hoxb13 delays neonatal CM cell cycle arrest (Nguyen et al. 2020).

#### TNNI3K

Tnni3k encodes a CM-specific kinase that predicts natural variation in CM ploidy in mice. Its knockout results in increased diploid CM content in the mouse myocardium and enhanced CM proliferation after infarction, while its overexpression in zebrafish increases CM ploidy in and impairs their cardiac regenerative response (Patterson et al. 2017). As such, Tnni3k may interrupt the cell cycle prior to cytokinesis in CMs, resulting in polyploidization.

#### LMNB2

Nuclear lamina filament Lamin B2 (Lmnb2) expression decreases after birth coinciding with the closure of the neonatal regenerative window in mice. It enables metaphase progression and its suppression increased CM polyploidization and reduced cardiac regeneration in neonatal mice (Han et al. 2020).

### Neovascularization & inflammation

#### CXCL12

Unlike P1 mice, P7 mice fail to regenerate blood vessels and form collateral arteries to bypass the ligation in the coronary artery after myocardial infarction. Cxcl12 is expressed primarily in the arterial endothelium in uninjured neonatal hearts, but is quickly induced by hypoxia in the endothelial cells of capillaries between coronary arteries after myocardial infarction. Deletion of Cxcl12 from cardiac endothelial cells compromised collateral artery formation and myocardial regeneration after injury, as did deletion of Cxcl12 receptor Cxcl4 from the arterial endothelium specifically. Conversely, exogenous Cxcl12 injection in between coronary arteries of infarcted adult hearts stimulated collateral artery formation as in the neonate (Das et al. 2019).

#### TLR

Toll-like receptor (TLR) signaling is upregulated in the regenerative zebrafish compared to the non-regenerative medaka after cardiac injury. TLRs sense broad damage-associated molecular patterns such as inflammatory cytokines and recruit immune cells to injury sites. Injection of TLR agonist poly I:C accelerated macrophage recruitment and neutrophil clearance in the injured medaka heart and resulted in increased CM proliferation and reduced fibrosis (Lai et al. 2017).

#### MMP3

As with neonatal mice, macrophage depletion blocks cardiac regeneration in axolotl salamanders. Matrix metalloproteinase 3 (MMP3) activates β-catenin signaling and induces epithelial-to-mesenchymal transition; it is significantly downregulated after macrophage depletion (Godwin et al. 2017).

#### IL-6

Interleukin-6 (IL-6) is the most upregulated cytokine after apical resection in P1 mice. Its knockout reduces post-injury CM proliferation and inhibits cardiac regeneration in P1 mice. Indeed, exogenous IL-6 injection alone induces CM proliferation in the uninjured P1 heart. The reactive CM proliferation induced by IL-6 is mediated through downstream effector STAT3, which is also required for neonatal CM regeneration (Han et al. 2015).

## Conclusion

**Fig. 1:**
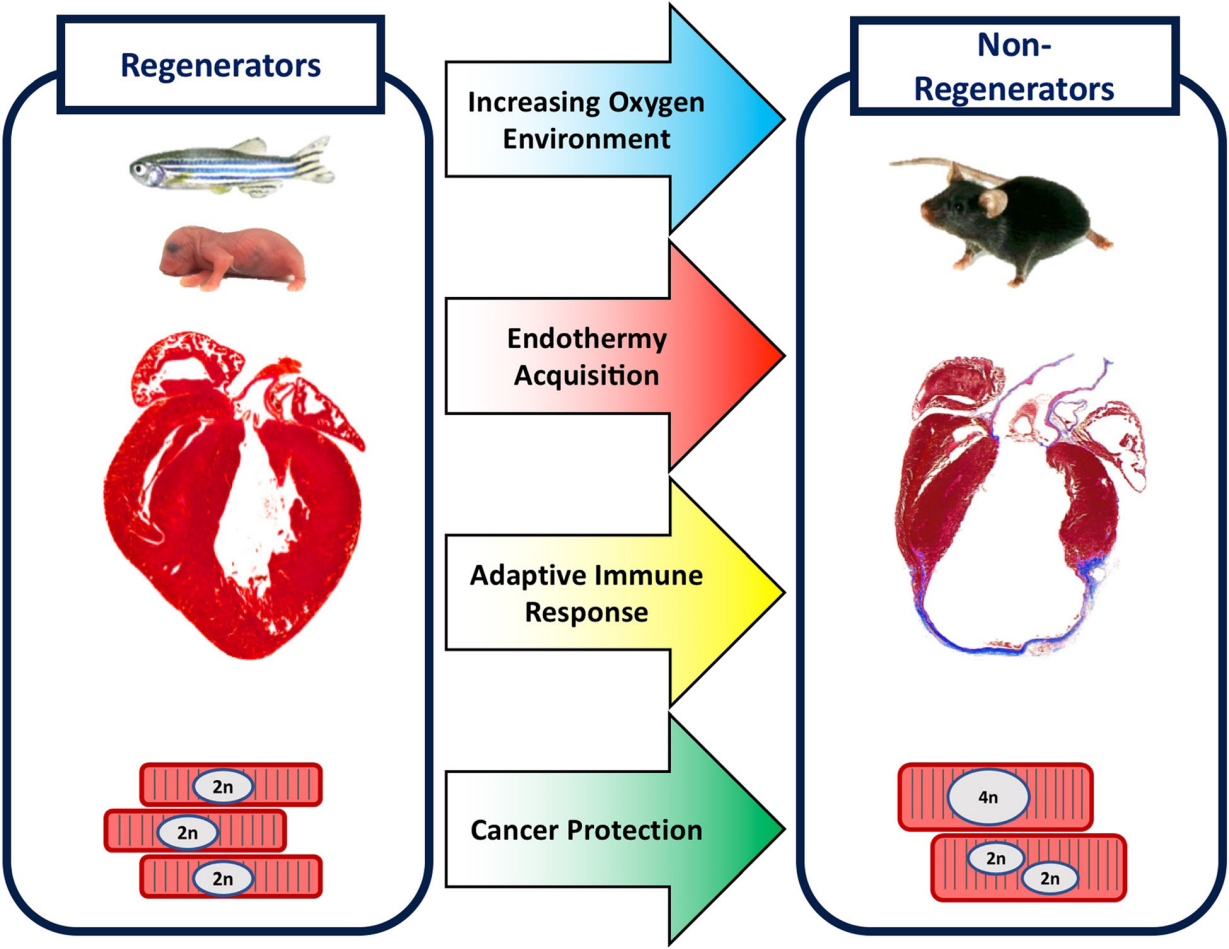
Visual representation of four major hypotheses of drivers of vertebrate cardiac regenerative potential loss in evolution and development

Although vertebrate cardiac regenerative potential varies both evolutionarily across different species and developmentally throughout the life of a given species, certain common themes still emerge. Broadly, cardiac regenerative potential has decreased throughout vertebrate evolution, with more ancestral lineages such as fish and amphibians generally displaying a greater capacity to regenerate the heart than more recent lineages like mammals. The increased oxidative stresses on adults and higher mammalian lineages, more efficient thermogenesis in adult endotherms, evolution and maturation of a robust adaptive immune response, and selective pressure to mitigate cancer risks have all been implicated in this loss of heart regeneration in adult higher vertebrates compared to their fetal stages and to lower vertebrates (Fig. 1). Mechanistically, effects have been exerted in part through several nuclear hormone receptors, metabolic regulators, cell cycle genes, and inflammatory and angiogenic factors.

As further research is conducted into the mechanisms that promote or inhibit vertebrate cardiac regenerative potential and the forces that act on these mechanisms, both our collective understanding of heart regeneration and our toolkit for promoting it therapeutically will expand. Based on the state of the field, valid directions to explore in the near future include probing into the mechanisms that shut down cardiac regeneration in certain species within otherwise regeneration-capable lineages (such as in medaka within teleost fish), as well as comprehensively assessing the cardiac regenerative potential of adult mammals that may induce CM proliferation after injury – such as bats. Additionally, identifying other physiological and molecular triggers that developmentally inhibit cardiac regenerative potential would elucidate how oxygen environment, endothermy acquisition, immune responses, and cancer risks all interplay to preclude heart regeneration – and specifically whether any factors dominate the others.
